# Autoantibodies Against Lysosome Associated Membrane Protein-2 (LAMP-2) in Pediatric Chronic Primary Systemic Vasculitis

**DOI:** 10.3389/fimmu.2020.624758

**Published:** 2021-02-03

**Authors:** Kristen M. Gibson, Renate Kain, Raashid A. Luqmani, Colin J. Ross, David A. Cabral, Kelly L. Brown

**Affiliations:** ^1^ Department of Medical Genetics, University of British Columbia, Vancouver, BC, Canada; ^2^ BC Children’s Hospital Research Institute, Vancouver, BC, Canada; ^3^ Department of Pathology, Medical University of Vienna, Vienna, Austria; ^4^ Nuffield Department of Orthopedics, Rheumatology and Musculoskeletal Sciences, University of Oxford, Oxford, United Kingdom; ^5^ Faculty of Pharmaceutical Sciences, University of British Columbia, Vancouver, BC, Canada; ^6^ Department of Pediatrics, University of British Columbia, Vancouver, BC, Canada; ^7^ Division of Rheumatology, BC Children’s Hospital, Vancouver, BC, Canada; ^8^ Centre for Blood Research, University of British Columbia, Vancouver, BC, Canada

**Keywords:** anti-neutrophil cytoplasmic antibody, ANCA-associated vasculitis, LAMP-2, lysosome-associated membrane protein-2, pediatric, systemic vasculitis

## Abstract

**Background:**

Anti-neutrophil cytoplasmic antibody (ANCA)-associated vasculitis (AAV) is a small vessel vasculitis in adults and children that commonly affects the kidneys. Although the frequent antigenic, and presumed pathogenic, targets of ANCA in AAV are proteinase-3 (PR3) and myeloperoxidase (MPO), ANCA against lysosome associated membrane protein-2 (LAMP-2), a lesser known ANCA antigen that is expressed on the glomerular endothelium, are present in some adults with AAV-associated renal disease. LAMP-2-ANCA has not been assessed in children with chronic systemic vasculitis, and, if present, would be a potentially valuable biomarker given that treatment decisions for these pediatric patients at diagnosis are largely informed by kidney function.

**Methods:**

A custom ELISA, using commercially available reagents, was designed to detect autoantibodies to human LAMP-2 in serum. Sera obtained from 51 pediatric patients at the time of diagnosis of chronic primary systemic vasculitis (predominantly AAV) were screened. LAMP-2-ANCA titers were evaluated for correlation with clinical metrics of disease activity (pediatric vasculitis activity score [pVAS], C-reactive protein [CRP] concentration, and erythrocyte sedimentation rate [ESR]), MPO- and PR3-ANCA titers, and renal function (glomerular filtration rate [GFR], renal-specific pVAS, and serum creatinine concentration).

**Results:**

LAMP-2-ANCA (>1,000 ng/ml) were detected in 35% (n = 18) of pediatric systemic vasculitis patients, of which, 10 (20% of all patients) were found to have high positive titers (>1,500 ng/ml). Undetectable or negative titres (<500 ng/ml) were identified in 12% (n = 6) of patients, those with titers between 500 and 1,000 ng/ml were considered low with unknown clinical relevance (53%, n = 27). Although LAMP-2-ANCA titers did not significantly differ between patients with AAV versus ANCA-negative vasculitis, only AAV patients had high concentrations (>1,500 ng/ml) of LAMP-2-ANCA. LAMP-2-ANCA titers did not correlate with measures of disease activity (pVAS, CRP, or ESR) at the time of diagnosis. In contrast, for patients with 12-month post diagnosis follow-up, a negative correlation was observed between the change in GFR (from diagnosis to 12-month follow-up) and LAMP-2-ANCA titer at diagnosis.

**Conclusions:**

Moderate to high LAMP-2-ANCA titers were detected in 35% (18/51) of children with chronic systemic vasculitis affecting small-to-medium vessels. Although the highest concentrations of LAMP-2-ANCA in this population were observed in individuals positive for classic ANCA (MPO- or PR3-ANCA), similar to previous reports on adult patients, LAMP-2-ANCA titers do not correlate with classic ANCA titers or with overall disease activity at diagnosis. Renal disease is a common manifestation in systemic small-medium vessel vasculitis (both in adults and children, though more severe in children) and our preliminary data suggest LAMP-2-ANCA at diagnosis may be a risk factor for more severe renal disease.

## Introduction

Anti-neutrophil cytoplasmic antibodies (ANCA) are a family of autoantibodies that are reactive against multiple proteins that are predominantly contained within intracellular granules of neutrophils (1, 2). These autoantibodies were first observed in individuals with glomerulonephritis (3) and forms of systemic small vessel vasculitis (4, 5) that were subsequently named ANCA-associated vasculitis (AAV). In AAV, there are two major classes of ANCA that are defined by the antigenic target: PR3-ANCA directed against proteinase-3 (PR3) and MPO-ANCA directed against myeloperoxidase (MPO). PR3-ANCA and MPO-ANCA are predominantly, but not exclusively, associated with different AAV subtypes (respectively, granulomatosis with polyangiitis and microscopic polyangiitis), and are used clinically to aid phenotype classification. More recently, the presence and specificity (for PR3 or MPO) of ANCA have helped to define disease-associated risks in adult AAV subtypes that do not overlap with the phenotypic classification (6). For example, patients positive for PR3-ANCA often have a more relapsing disease course, increased risk of severe inflammatory lung disease, and systemic disease involving multiple organs at diagnosis (7, 8). In contrast, MPO-ANCA positive patients are more likely to have more severe renal-limited disease (9, 10). Some data on adult patients also supports the value of serially measuring ANCA titers as a marker of disease activity (7, 11), but whether ANCA are informative to organ-specific disease processes, which is a primary determinant in treatment decisions, remains to be shown. Although there is a high incidence of kidney disease in AAV, MPO and PR3 are not expressed by the glomerular endothelium, the primary site of injury in patients with renal involvement (10). Although MPO and PR3 released by neutrophils may associate with the endothelium and in this manner target the endothelium for ANCA-mediated damage, it is also possible that disease processes are the result of the indirect action of ANCA, or are independent of autoantibodies, as may be the case in patients with ANCA-negative vasculitis (2).

A search for autoantigenic targets expressed on the membrane of glomerular cells that may serve as a more direct target of autoimmune processes led to the discovery by Kain et al. in 1995 (12) of antibodies against lysosome associated membrane protein-2 (LAMP-2/CD107b). These LAMP-2-ANCA were detected in adults with active necrotising and crescentic glomerulonephritis (12) and whom were also frequently positive for PR3-ANCA or MPO-ANCA. It was further demonstrated that one of the most common ANCA recognition epitopes on LAMP-2 has 100% homology with the Type I fimbriated bacterial adhesion protein, FimH. Notably, FimH-immunized rats developed pauci-immune focal necrotizing glomerulonephritis and ANCA to both rat and human LAMP-2 (13). Despite this *in vivo* evidence of LAMP-2-ANCA pathogenicity and subsequent findings of LAMP-2-ANCA in cohorts of adults with small-to-medium sized vessel vasculitis (12–15), other studies demonstrate similar LAMP-2-ANCA titers in healthy individuals and patients (16). These contradictory findings may reflect the absence of a standardized assay for LAMP-2-ANCA, impact of immunosuppressive therapy on ANCA titers, and patient selection criteria (17, 18).

The prevalence of LAMP-2-ANCA has not been assessed in children with vasculitis due in large part to the rarity of the disease relative to adult-onset vasculitis. The aim of this study was to conduct a preliminary screen of a retrospective collection of sera from pediatric patients with small-to-medium vessel chronic primary systemic vasculitis for the presence of LAMP-2-ANCA. Without a commercially available assay for LAMP-2-ANCA, we designed a custom enzyme-linked immunosorbent assay (ELISA) and quantified the concentration of LAMP-2-ANCA in sera from 51 pediatric vasculitis patients at the time of diagnosis. Our findings demonstrate that LAMP-2-ANCA are present in children with systemic vasculitis and provide preliminary evidence that LAMP-2-ANCA titers at the time of diagnosis can indicate worse renal outcomes.

## Materials and Methods

### Pediatric Patients, Clinical Data, and Samples

Patients described in this study were enrolled in the Pediatric Vasculitis Initiative (PedVas), an international study on chronic primary systemic vasculitis in children. Eligibility criteria for PedVas have been described previously (19). The study protocol was approved by the Children’s and Women’s Research Ethics Board of the University of British Columbia [H12-00894] and the respective ethical committees or IRBs at participating PedVas sites. At the time of diagnosis, participating centres collected sera and clinical data (including, but not limited to, positivity for PR3-ANCA and MPO-ANCA, and glomerular filtration rate) as described (20). Using entered information from participating sites, patients were formally classified into small-to-medium vasculitis subtypes using a pediatric modified algorithm of the European Medicines Agency (EMA) (21). Disease activity at the time of sample collection was calculated using the pediatric vasculitis activity score (pVAS) (22). Pediatric inflammatory disease controls included five patients diagnosed with an autoinflammatory disease/periodic fever syndrome and followed at the BC Children’s Hospital, Vancouver, BC. All inflammatory controls were enrolled in a research study approved by Children’s and Women’s Research Ethics Board of the University of British Columbia [H15-00351]. All participants (pediatric vasculitis patients and autoinflammatory controls) contributed blood in K_2_EDTA and/or serum separation tubes (both from BD Biosciences, NJ, USA) during a flare in disease. Blood was processed to serum and plasma according to standard protocols from the manufacturer and aliquots were stored at −80°C.

### Enzyme-Linked Immunosorbent Assays

Concentrations of C-reactive protein (CRP) were measured in sera using a human CRP ELISA kit (ThermoFisher, MA, USA) according to manufacturer’s instructions (23). Concentrations of PR3-ANCA (ORG518, Orgentec) and MPO-ANCA (425–2380, BioRad) were measured according to manufacturer’s instructions and as described previously (23). Concentrations of LAMP-2-ANCA were measured by a custom indirect ELISA (**Supplementary Figure S1**) as follows: Nunc MaxiSorp™ flat-bottom 96-well plates (ThermoFisher, MA, USA) were coated with 50 µl of 5 µg/ml recombinant human (rh) LAMP-2 protein (R&D Systems, MN, USA) diluted in 0.2 M carbonate-bicarbonate, pH 9.4 coating buffer (ThermoFisher, MA, USA), and incubated overnight at 4°C. Wells were washed 3× with 250 µl/well wash buffer (WB; PBS containing 0.05% Tween^®^20 [FisherScientific, MA, USA]). Blocking buffer (BB; PBS containing 0.05% Tween^®^20 and 2% bovine serum albumin [MilliporeSigma, MA, USA]) was added (300 µl/well) and incubated at room temperature (RT) for 1 h. BB was discarded and standards/samples were added without washing the plate. Sample (serum or plasma) was diluted 1/10 in BB. Detection reagents were prepared in PBS containing 0.01% Tween^®^20 and 0.4% BSA. Standards were generated using anti-human LAMP-2 monoclonal antibody (H4B4) (Invitrogen, CA, USA) serially diluted in BB, with optimal dilution range of 50–1,000 ng/ml. Diluted standards and samples were plated (100 µl/well) in duplicate and incubated at RT for 1 h. Wells were washed 5× with 300 µl/well WB then incubated 1 h at RT with 100 µl/well of 1 µg/ml CaptureSelect™ biotin anti-IgG-Fc (multi-species) conjugate (ThermoFisher, MA, USA). Wells were washed 5× with 300 µl/well WB then incubated at RT 30 min with 100 µl/well of 0.5 µg/ml horseradish peroxidase (HRP)-conjugated streptavidin (ThermoFisher, MA, USA). Tetramethylbenzidine (TMB) substrate solution (ThermoFisher, MA, USA) was added (100 µl/well) and incubated for 30 min at RT. TMB stop solution (ThermoFisher, MA, USA) was added 50 µl/well, and absorbance read on the Tecan Infinite M200 spectrophotometer (Tecan, Switzerland) at 450 nm, with a reference read at 620 nm. By fitting the standard curve to a sigmoidal, 4 parameter logistic regression (4PL) equation, unknown values with an absorbance (Abs) at 450 nm (Abs_450_) were interpolated between 0.402 AU (lower-limit) and 2.776 AU (upper-limit). Optimal sera dilution was found to be 1/10 (data not shown). The ELISA was validated with human sera from young-onset AAV patients, previously reported to be positive (n = 5) or negative (n = 1) for LAMP-2-ANCA at the Medical University of Vienna (13, 14).

### Statistical Analysis

Statistical analyses were done using GraphPad Prism v8.0 Statistical Software (GraphPad Software, CA, USA). Group differences were analyzed by ANOVA and subsequent two-tailed t-tests. Correlations were assessed by Pearson correlation coefficient. For all analyses, a confidence interval of 95% was used; a p-value < 0.05 was considered significant.

## Results

### LAMP-2-ANCA Are Present in Children With Chronic Systemic Small-Medium Vasculitis

**Figure 1:**
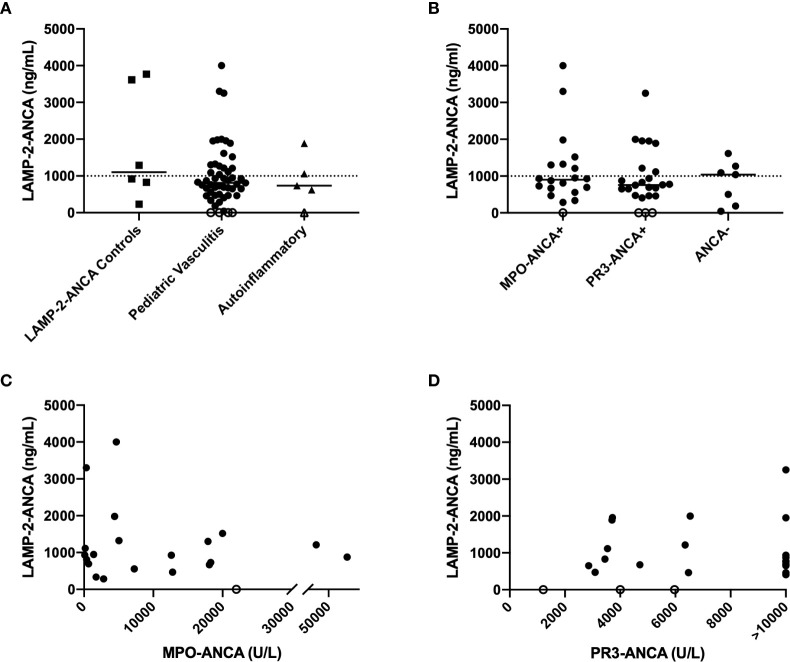
Concentration of LAMP-2-ANCA in pediatric chronic small-to-medium vessel vasculitis patients. **(A)** LAMP-2-ANCA concentration (y-axis; ng/mL) in serum of individuals with early-onset vasculitis that are known to be positive (n = 5) and negative (n = 1) for LAMP-2-ANCA (squares) (14), children with vasculitis (n = 51, circles), and children with systemic autoinflammatory disease (n = 5, triangles). **(B)** LAMP-2-ANCA concentration (y-axis; ng/mL) in pediatric patients grouped (x-axis) based on positivity for MPO-ANCA (n = 19), PR3-ANCA (n = 23), or neither MPO- or PR3-ANCA (ANCA-, n = 7), and **(C, D)** LAMP-2-ANCA concentration (y-axis; ng/mL) plotted against **(C)** MPO-ANCA (x-axis; U/L) (n =19) and **(D)** PR3-ANCA (x-axis; U/L) (n = 23). Bars show median. Horizontal line divided low (<1,000 ng/mL) and moderate-high positive LAMP-2-ANCA (>1,000 ng/mL). Open symbols on the x-axis denote samples below the lower limit of detection of the assay (n = 4 patients with vasculitis, and n = 1 patient with autoinflammatory disease).

A custom ELISA (described in methods and **Supplementary Figure S1**) was designed to determine if LAMP-2-ANCA are present in sera obtained from children with systemic vasculitis affecting small-to-medium sized vessels, the most common of which is ANCA-associated vasculitis (AAV). The ELISA was validated with human sera from individuals with early-onset AAV and known to be positive (n = 5, with high titers in two samples and moderate-low titers in three samples) and negative (n = 1) for LAMP-2-ANCA (13, 14). Concentrations of LAMP-2-ANCA in these samples as determined by the ELISA were as expected; the negative sample contained the lowest calculated concentration (229.8 ng/ml) of LAMP-2-ANCA, and titers in the low-moderate to high-positive samples ranged from 828.3–3,768.95 ng/ml (**Figure 1A**). Sera from five children with systemic inflammation due to an autoinflammatory disease were screened as controls; LAMP-2-ANCA concentration in 4/5 samples were on the lower end of the positive range (<1,062 ng/ml) (**Figure 1A**). Using these interpolated measures and for the purpose of this study, we reasoned that LAMP-2-ANCA concentrations <250 ng/ml would be considered negative and <1,000 ng/ml were low titers with unknown clinical relevance. Titers >1,000 ng/ml were considered positive with high titer measuring >1,500 ng/ml.

Using these established boundaries, 51 pediatric patients diagnosed with chronic primary systemic vasculitis affecting small-to-medium sized vessels (**Table 1A**) were screened for the presence of LAMP-2-ANCA. Of these, 19 patients were positive for MPO-ANCA, 23 were positive for PR3-ANCA, one patient had both MPO- and PR3-ANCA, and eight patients were ANCA-negative. The mean age of onset of disease was 12.6 years, and the ratio of males to females was equally distributed between groups. Similar to LAMP-2-ANCA-positive control sera, LAMP-2-ANCA concentrations in pediatric vasculitis samples ranged from undetectable (n = 4 patients) to levels over 3 µg/ml (n = 3 patients) (**Figure 1A**). Overall, 12% (n = 6) had undetectable or negative (< 250 ng/ml) LAMP-2-ANCA and 53% (n = 27) were found to have low titers (<1,000 ng/ml) of unknown clinical significance. The remaining 35% (n = 18) of pediatric vasculitis patients had a minimum of 1,000 ng/ml of LAMP-2-ANCA, with 56% of those individuals (and 20% of total patients) having high-positive titers (>1,500 ng/ml).

**Table 1A:** Pediatric vasculitis cohort.

ID	Diagnosis^a^	Onset^b^	Sex	ANCA	Renal^c^	Treatment^d^
**1**	uAAV	16	Male	MPO	No	prednisone
**2**	MPA	15	Female	MPO	Yes	prednisone
**3**	GPA	12	Female	MPO	Yes	none
**4**	GPA	11	Female	MPO	Yes	prednisone
**5**	GPA	12	Female	MPO	No	prednisone, methotrexate
**6**	UCV	17	Female	MPO	Yes	prednisone, cyclophosphamide
**7**	GPA	15	Male	MPO	Yes	none
**8**	GPA	5	Female	MPO	Yes	prednisone, methotrexate, rituximab, azathioprine
**9**	GPA	16	Female	MPO	Yes	none
**10**	GPA	2	Male	MPO	Yes	none
**11**	GPA	13	Female	MPO	Yes	none
**12**	GPA	17	Female	MPO	Yes	prednisone, cyclophosphamide, rituximab
**13**	GPA	15	Male	MPO	Yes	prednisone
**14**	UCV	17	Female	MPO	Yes	none
**15**	MPA	16	Male	MPO	Yes	none
**16**	MPA	17	Female	MPO	Yes	cyclophosphamide
**17**	GPA	17	Male	MPO	Yes	prednisone, cyclophosphamide
**18**	GPA	1	Female	MPO	Yes	prednisone, rituximab
**19**	GPA	5	Female	MPO	Yes	prednisone, cyclophosphamide
**20**	MPA	5	Female	MPO	Yes	prednisone
**21**	GPA	10	Male	PR3	Yes	none
**22**	GPA	15	Female	PR3	Yes	none
**23**	GPA	13	Female	PR3	Yes	prednisone
**24**	GPA	15	Male	PR3	Yes	none
**25**	NA	17	Female	PR3	Yes	prednisone
**26**	GPA	12	Female	PR3	Yes	prednisone, rituximab
**27**	GPA	15	Male	PR3	Yes	prednisone
**28**	GPA	15	Female	PR3	Yes	prednisone, rituximab
**29**	uAAV	12	Female	PR3	No	prednisone, methotrexate
**30**	GPA	14	Male	PR3	No	prednisone, rituximab
**31**	GPA	14	Female	PR3	Yes	none
**32**	GPA	14	Female	PR3	Yes	prednisone, cyclophosphamide
**33**	uAAV	13	Male	PR3	Yes	prednisone
**34**	UCV	13	Female	PR3	Yes	prednisone, cyclophosphamide
**35**	GPA	12	Female	PR3	Yes	prednisone, cyclophosphamide
**36**	GPA	12	Female	PR3	Yes	prednisone, cyclophosphamide, rituximab
**37**	UCV	14	Female	PR3	Yes	prednisone
**38**	GPA	15	Female	PR3	Yes	prednisone, cyclophosphamide, rituximab
**39**	GPA	14	Female	PR3	Yes	prednisone, rituximab
**40**	GPA	15	Male	PR3	Yes	prednisone, rituximab
**41**	GPA	18	Male	PR3	Yes	prednisone, rituximab
**42**	GPA	16	Male	PR3	Yes	prednisone, rituximab
**43**	GPA	16	Male	PR3	yes	prednisone, rituximab
**44**	MPA	13	Male	Both	Yes	none
**45**	uAAV	10	Female	Negative	No	prednisone
**46**	uAAV	9	Male	Negative	No	prednisone
**47**	cPAN	2	Male	Negative	Yes	prednisone, infliximab
**48**	UCV	15	Female	Negative	Yes	prednisone
**49**	GPA	7	Female	Negative	Yes	none
**50**	UCV	4	Male	Negative	No	prednisone
**51**	PAN	16	Female	Negative	No	none

^a^According to the EMA classification algorithm (21). MPA, microscopic polyangiitis; GPA, granulomatosis with polyangiitis; PAN, polyarteritis nodosa; uAAV, unclassified ANCA-associated vasculitis, UCV, unclassified vasculitis. ^b^Age in years when symptoms associated with vasculitis first presented ^c^Renal involvement determined by renal component of the pVAS => 4 ^d^Current treatments at diagnosis, coincident with sample and data collection.

Although the highest concentrations of LAMP-2-ANCA were present in patients also positive for PR3-ANCA or MPO-ANCA (compared to ANCA-negative patients), LAMP-2-ANCA titers did not significantly differ between patients positive for the classic ANCA subsets (MPO-ANCA, PR3-ANCA) or ANCA-negative patients (n = 47, *p* = 0.5715) (**Figure 1B**). Moreover, within the subset of ANCA-positive patients, there was no correlation between LAMP-2-ANCA titers and titers (**Table 1B**) of either MPO-ANCA (n = 19, *p* = 0.6054, **Figure 1C**) or PR3-ANCA (n = 21, *p* = 0.9897, **Figure 1D**). No correlation was observed between LAMP-2-ANCA titer and age of onset or sex (data not shown).

**Table 1B:** Measures of disease activity, and LAMP-2-, PR3- and MPO-ANCA.

ID	pVAS^a^ ^,^ ^b^	CRP^b^ (mg/L)	ESR^b^ (mm/hr)	LAMP-2-ANCA^b^ (ng/mL)	MPO-ANCA (U/mL)	PR3-ANCA (U/mL)
**1**	10	19.7	1	1,301.1	17,896.5	–
**2**	12	20.0	1	948.6	1,392.1	–
**3**	19	8.0	127	1,323.7	5,034.3	–
**4**	20	3.7	150	668.3	18,087.8	–
**5**	10	3.4	17	468.0	12,777.2	–
**6**	25	9.7	9	729.5	18,284.9	–
**7**	22	5.3	44	875.9	68,007.5	–
**8**	17	32.6	100	4,000.5	4,660.0	–
**9**	14	722.4	61	1,520.0	19,978.4	–
**10**	20	15.1	78	33,02.7	324.2	–
**11**	31	23.5	90	932.5	109.4	–
**12**	30	129.2	107	555.9	7,241.3	–
**13**	21	244.7	nd^c^	809.1	364.0	–
**14**	18	23.4	nd^c^	693.9	663.1	–
**15**	16	163.3	130	ND^d^	21,974.3	–
**16**	21	14.3	100	337.1	1,746.9	–
**17**	20	6,332.1	104	1,979.9	4,404.1	–
**18**	19	38.7	nd^c^	284.7	28,25.2	–
**19**	16	122.9	nd^c^	928.1	12,609.5	–
**20**	14	313.6	140	1,208.4	38,083.8	–
**21**	20	23.9	16	1,950.2	–	>10000
**22**	19	272.8	38	3,250.6	–	44,685.8
**23**	32	219.9	96	1,890.3	–	3,700.4
**24**	38	2,560.2	nd^c^	692.3	–	>10000
**25**	18	8.0	23	ND^d^	–	12,13.5
**26**	31	7.8	9	874.1	–	14,263.1
**27**	21	7.3	nd^c^	460.0	–	550,151.0
**28**	21	7.2	18	678.0	–	4,713.3
**29**	7	80.4	26	464.8	–	6,472.5
**30**	21	116.3	36	405.6	–	>10000
**31**	30	11.4	72	777.6	–	>10000
**32**	23	8.6	115	653.8	–	>10000
**33**	21	79.7	140	758.6	–	>10000
**34**	nd^b^	13.8	95	831.2	–	3,444.7
**35**	21	9.2	170	1,212.6	–	6,351.7
**36**	19	28.6	120	937.2	–	10,196.8
**37**	23	24.8	nd^c^	434.9	–	>10000
**38**	50	249.2	130	652.2	–	2,858.3
**39**	33	6.4	40	ND^d^	–	5,970.7
**40**	33	82.7	110	1,997.9	–	6,533.4
**41**	31	472.6	70	474.4	–	3,089.1
**42**	28	731.9	53	1,954.8	–	3,718.1
**43**	20	7.6	98	ND^d^	–	4,001.1
**44**	15	163.4	54	1,115.9	173.4	3,542.3
**45**	6	103.6	17	1,614.2	–	–
**46**	5	70.8	87	186.3	–	–
**47**	7	21.8	68	45.1	–	–
**48**	15	790.2	15	1,266.8	–	–
**49**	17	962.5	97	501.4	–	–
**50**	12	128.4	78	1,089.7	–	–
**51**	9	5.3	85	1,039.6	–	–

a pVAS: pediatric vasculitis activity score.

b Measurement taken at time of diagnosis.

c nd, not done; ^d^ND, not detected (Abs_450_ below the lower limit of detection).

### LAMP-2-ANCA Titers Do Not Correlate With Clinical Disease Activity Measures

**Figure 2:**
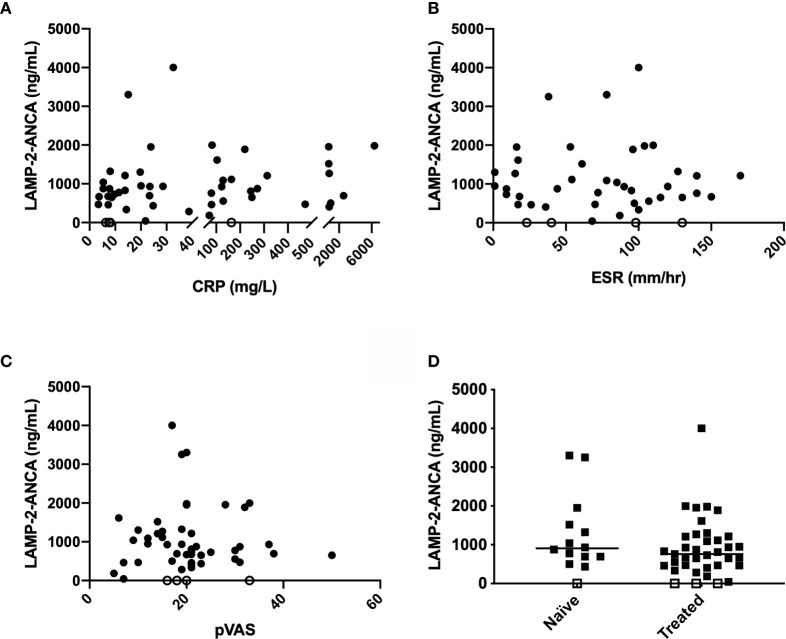
Comparison of LAMP-2-ANCA titer with standard clinical measures of disease activity. Concentration of LAMP-2-ANCA (y-axis; ng/mL) in pediatric vasculitis patients plotted against **(A)** C-reactive protein (CRP) concentration (x-axis; mg/L) (n = 51), **(B)** erythrocyte sedimentation rate (ESR) (x-axis; mm/h) (n = 44), and **(C)** pediatric vasculitis activity score (pVAS) (x-axis) (n = 51) at the time of diagnosis, and **(D)** blood samples taken prior to (naïve, n = 14), or after (treated, n = 37), induction of immune suppressive therapy. Bars show median. Horizontal line divided low (<1,000 ng/mL) and moderate-high positive LAMP-2-ANCA (>1000 ng/mL). Open symbols on the x-axis denote samples below the lower limit of detection of the assay (n = 4).

We next assessed whether concentrations of LAMP-2-ANCA correlated with standard clinical measures of disease activity, namely, C-reactive protein (CRP, mg/L), erythrocyte sedimentation rate (ESR, mm/hr), and pediatric vasculitis activity score (pVAS). ESR and pVAS were derived from clinical data entered at the participating site, and CRP was measured in house by commercial ELISA (see methods). Neither CRP (n = 47, *p* = 0.3115) nor ESR (n = 41, *p* = 0.9707) were found to correlate with LAMP-2-ANCA titers (**Figures 2A, B**). Likewise, LAMP-2-ANCA titers did not correlate with the pediatric vasculitis activity score (pVAS, **Figure 2C**) (n = 46, *p* = 0.9737), a pediatric adaption of the adult BVAS, which is a cumulative weighted score of disease activity of nine organ systems (mean pVAS = 20.4 +/- 8.8 at TOD, n = 46) (22). Consistent with these findings, LAMP-2-ANCA titers did not differ between samples collected prior to or shortly after immune suppressive induction therapy (n = 47, *p* = 0.2068) (**Figure 2D**).

### LAMP-2-ANCA Titers Are Associated With Worsening Renal Disease

Both adult and pediatric ANCA-associated vasculitides are frequently associated with kidney disease. In our cohort, eighty-four percent (n = 43) of patients screened for LAMP-2-ANCA had renal involvement, as determined by the renal component of the pVAS being ≥ 4 (**Tables 1A**, **C**). The renal component of the pVAS takes into account renal hypertension, glomerular filtration rate (GFR), and the presence of hematuria, RBC casts, and proteinuria. Mean renal pVAS at the TOD was 11.4 +/- 8.3 (n = 50). As observed for overall score of disease activity (total pVAS, **Figure 2C**), no correlation between LAMP-2-ANCA and renal pVAS was observed (n = 46, *p* = 0.9734; data not shown).

**Table 1C:** Renal metrics.

ID	Renal pVAS^a^	GFR (mL/min/1.73m^2^)	
		TOD^b^	12-month
**1**	0	119	97
**2**	28	9	85
**3**	26	20	34
**4**	20	56	96
**5**	0	nd^c^	nd^c^
**6**	20	nd^c^	nd^c^
**7**	14	nd^c^	nd^c^
**8**	10	nd^c^	nd^c^
**9**	4	102	93
**10**	4	99	nd^c^
**11**	28	2	nd^c^
**12**	22	nd^c^	nd^c^
**13**	14	53	64
**14**	28	nd^c^	nd^c^
**15**	10	54	62
**16**	12	8	nd^c^
**17**	12	4	nd^c^
**18**	12	3	nd^c^
**19**	12	6	27
**20**	12	13	58
**21**	6	94	nd^c^
**22**	16	91	10
**23**	22	25	7
**24**	26	26	6
**25**	6	nd^c^	nd^c^
**26**	6	182	148
**27**	6	146	130
**28**	10	112	90
**29**	0	nd^c^	nd^c^
**30**	0	nd^c^	nd^c^
**31**	10	nd^c^	nd^c^
**32**	10	nd^c^	nd^c^
**33**	10	121	67
**34**	24	0	62
**35**	10	147	113
**36**	24	5	22
**37**	10	97	94
**38**	12	80	nd^c^
**39**	10	133	93
**40**	12	142	108
**41**	12	10	nd^c^
**42**	12	49	nd^c^
**43**	12	7	8
**44**	14	nd^c^	nd^c^
**45**	0	136	111
**46**	0	nd^c^	nd^c^
**47**	4	nd^c^	nd^c^
**48**	4	nd^c^	115
**49**	6	148	nd^c^
**50**	0	129	111
**51**	0	127	nd^c^

a Renal pVAS score at time of diagnosis.

b TOD, time of diagnosis.

c nd, not done.

**Figure 3:**
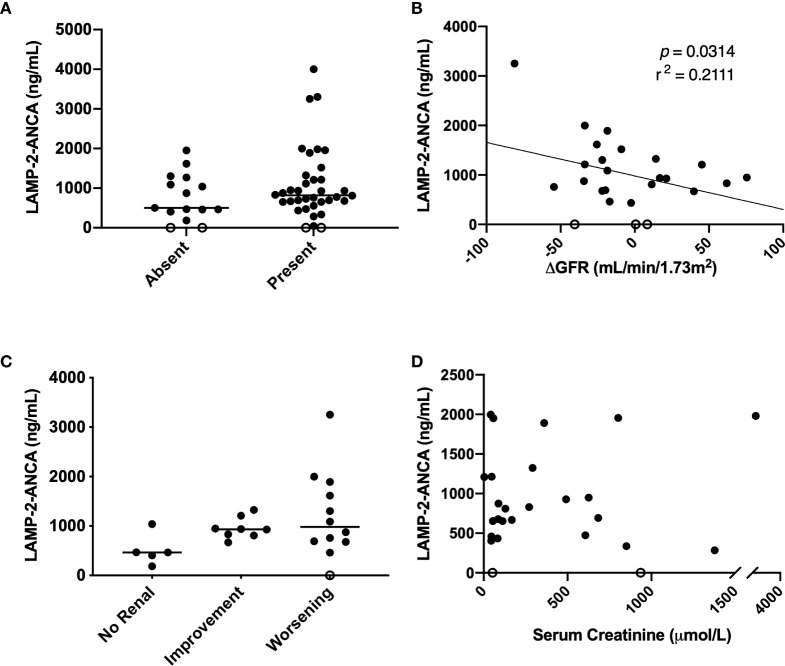
Comparison of LAMP-2-ANCA titer with renal metrics. Concentration of LAMP-2-ANCA (y-axis: ng/ml) in pediatric vasculitis patients: **(A)** with the absence (n = 15) and presence (n = 36) of proteinuria (x-axis) at the time of diagnosis. **(B)** plotted against change in GFR from the time of diagnosis to 12-month follow-up (x-axis, ml/min/1.73m^2^) (n = 25, *p* = 0.0314). **(C)** with no renal involvement (renal PVAS < 4; n = 5), and either renal improvement (increase in GFR at 12-month > 10 ml/min/1.73m^2^, n = 8) or worsening (decrease in GFR at 12-month > 10 ml/min/1.73m^2^, n = 12) from diagnosis to 12-month follow-up. **(D)** plotted against serum creatinine concentration (x-axis; µmol/L) (n = 26, *p* = 0.2149). Bars show median.

We next looked for correlations with individual indicators of renal function: proteinuria, GFR, and serum creatinine concentration. While no significant difference was observed, the presence of proteinuria was found in all patients with high LAMP-2-ANCA titers at the time of diagnosis (**Figure 3A**). Similarly, no significant correlation was observed between GFR at the time of diagnosis (**Table 1C**) and LAMP-2-ANCA titers (r^2^ = 0.0164, *p* = 0.4767, n = 33; data not shown). In adults with ANCA-associated vasculitis, poor renal outcomes are associated with a negative change in GFR at 12 months (24), where negative values indicate a decrease in kidney function. For a subset of our pediatric patients (n = 27) that had follow-up clinical data, we also observed a negative correlation between the change in GFR (from time of diagnosis to 12-month follow-up) and LAMP-2-ANCA titers (r^2^ = −0.2111, *p* = 0.0314) (**Figure 3B**). Similarly, there is a trending increase in LAMP-2-ANCA titers in patients with worsening renal disease at 12 months, as determined by a decrease in GFR > 10 ml/min/1.73m^2^ (**Figure 3C**). As serum creatine concentration at disease onset has been shown to be a risk factor for end stage renal disease (10, 25), the correlation with LAMP-2-ANCA was assessed, however, no correlation was observed (n = 24, r^2^ = 0.0625, *p* = 0.2387) (**Figure 3D**).

## Discussion

ANCA positivity and specificity for either PR3 or MPO aids phenotype classification in adult and pediatric AAV, and in adult-onset AAV is associated with general features of disease course (7–10). Their utility as prognostic markers for renal disease, which has a high prevalence among patients with AAV, may have limitations given that MPO and PR3 are not expressed on the glomerular endothelium. Unlike MPO or PR3, a lesser known ANCA antigen, LAMP-2, is expressed on the surface of the renal microvascular endothelium and LAMP-2-ANCA have been detected in adults with AAV-associated renal disease. The prospect of evaluating LAMP-2-ANCA for direct role(s) in the pathogenesis of renal disease associated with vasculitis or as a biomarker of glomerular damage (13) is inviting, particularly in children with AAV that, compared to adult-onset disease, present with more severe disease involving multiple organs (19, 26) and more than half of patients experience kidney damage early in disease course (27).

Herein, we conducted a preliminary screen of time of diagnosis sera from children (n = 51) with primary systemic small-to-medium sized vessel vasculitis (predominantly AAV) for the presence of LAMP-2-ANCA. Using a custom, in-house indirect ELISA, our data demonstrate that LAMP-2-ANCA are present in pediatric vasculitis patients. The majority of individuals were positive for low levels of LAMP-2-ANCA (53%), the clinical utility of which is unknown. An additional 35% of patients in the cohort had moderate-high titers of LAMP-2-ANCA (>1,000 ng/ml) and the remaining 12% of patients were negative for LAMP-2-ANCA. LAMP-2-ANCA titers did not correlate with positivity (or lack thereof) or titers of the classic PR3-ANCA and MPO-ANCA. LAMP-2-ANCA titers were also not correlated with elevated systemic disease activity as indicated by a validated pediatric vasculitis clinical scoring algorithm, pVAS, and general inflammatory markers, CRP and ESR. LAMP-2-ANCA titers may however be informative of renal function, which is affected in the majority of patients (84% in this cohort). Increasing LAMP-2-ANCA titers were observed in patients with declining glomerular filtration rate (GFR), indicative of worsening renal disease one-year post diagnosis.

Within the cohort, 88% of patients were positive for LAMP-2-ANCA with titers for the majority overlapping with concentrations detected in a control (autoinflammatory) cohort. Titers in the moderate to high range (>1,000 ng/ml), that, arguably, have a higher likelihood of disease association, were identified in 35% of patients in the cohort. The number of patients in our cohort with “moderate-high titer” positivity falls between conflicting rates of LAMP-2-ANCA positivity reported in two independent cohorts of adults with AAV, ranging from 21% (16) to >80% positivity for LAMP-2-ANCA (14). As summarized previously (18), these variable prevalence rates could be due to characteristics of the individual cohorts or assays used to assess LAMP-2-ANCA concentration. LAMP-2-ANCA titers are highly sensitive to immunosuppressive therapy, decreasing rapidly following treatment induction (14). As may be expected, higher prevalence rates of LAMP-2-ANCA were observed in patients with active disease and not on treatment. While the majority of the pediatric patients assayed in our study were not treatment naïve, samples were drawn early in disease course when disease activity was high. This may explain why LAMP-2-ANCA titers in our cohort were not significantly higher in the subset of treatment naïve patients.

In the highest reported prevalence rate of LAMP-2-ANCA in >80% of adults with AAV-associated renal disease, a recombinant, non-glycosylated human LAMP-2 protein was utilized in the immunoassays (14). While patient derived LAMP-2-ANCA have previously been shown to bind epitopes within non-glycosylated sites of the protein backbone (12, 13), non-human mammalian protein expression systems, such as the mouse myeloma line used to produce the rhLAMP-2 used in the described ELISA, may induce glycosylation patterns not found in humans (18). This potentially apparent glycosylation of LAMP-2 could block the endogenous LAMP-2-ANCA epitope — another possible explanation to the varying prevalence rate of LAMP-2-ANCA observed in our pediatric cohort compared to other cohorts.

Reported prevalence rates are also dependent on where the positive and negative thresholds are drawn. While LAMP-2-ANCA were detected in 88% of our cohort of pediatric vasculitis samples, the majority were deemed low titers (<1,000 ng/ml). Low LAMP-2-ANCA titers were also observed in pediatric autoinflammatory controls, with one control having a high titer (>1,500 ng/ml). The observation of high LAMP-2-ANCA in a disease control cohort is similar to previous reports, where LAMP-2-ANCA were detected in 10 - 16% of disease controls (14, 16). These results are not unexpected, as it’s not uncommon to detect autoantibodies in otherwise healthy individuals (28). In particular, given the molecular mimicry hypothesis (13), an individual with a previous Type I fimbriated bacterial infection could theoretically develop antibodies to LAMP-2.

The presence of LAMP-2-ANCA in some healthy individuals augments the importance of determining clinical utility of these autoantibodies. This can be difficult for rare populations, such as pediatric vasculitis, but our preliminary data suggest that, despite the lack of a correlation with markers of systemic disease activity (pVAS, CRP, ESR), LAMP-2-ANCA titers at diagnosis were negatively correlated with the change in GFR (from diagnosis to 12-months), a marker of renal function. As well, there was a trending increase in LAMP-2-ANCA at diagnosis in patients with worsening renal involvement at 12-month follow-up—patients with higher LAMP-2-ANCA at diagnosis, generally had worsening renal function after 12-months. Although sample numbers are a limitation in our study, these data suggest that LAMP-2-ANCA titers have potential utility as predictive marker of renal outcome.

In summary, a custom ELISA was designed to detect LAMP-2-ANCA in serum. This ELISA was used to screen a cohort of pediatric patients with AAV, to assess, for the first time, if LAMP-2-ANCA are prevalent in pediatric vasculitis. While LAMP-2-ANCA showed no correlation with MPO- or PR3-ANCA or markers of disease activity, evidence suggests a possible role for LAMP-2-ANCA as a predictive marker for renal outcome. As renal disease is a common manifestation in both children and adults with systemic small-medium vessel vasculitis, and often more severe in children, a prognostic biomarker could be invaluable to help guide effective treatment. Screening of a larger pediatric cohort with detailed follow-up will be necessary to elucidate the role of LAMP-2-ANCA in renal outcomes in children with chronic systemic vasculitis.

## Data Availability Statement

The raw data supporting the conclusions of this article will be made available by the authors, without undue reservation.

## Ethics Statement

The studies involving human participants were reviewed and approved by Children’s and Women’s Research Ethics Board of the University of British Columbia. Written informed consent to participate in this study was provided by the participants’ legal guardian/next of kin.

## Author Contributions

KG, RK, RL, CR, DC, and KB contributed to conception and design of the study. RK provided sera controls for LAMP-2-ANCA. KG acquired data and performed the statistical analyses. All authors contributed to the article and approved the submitted version.

## Funding

This study has been supported by a Canadian Institutes of Health Research grant for the PedVas Initiative [TR2-119188 to DC]. KB and CR are supported by Michael Smith Foundation for Health Research Awards. KG is supported by the University of British Columbia Four Year Fellowship for PhD Students. DC is supported by The Arthritis Society (TAS) Canada through the Ross Petty Arthritis Society Chair.

## Conflict of Interest

The authors declare that the research was conducted in the absence of any commercial or financial relationships that could be construed as a potential conflict of interest.

## Abbreviations

AAV, ANCA-associated vasculitis; ANCA, anti-neutrophil cytoplasmic antibody; GN, glomerulonephritis; GPA, granulomatosis with polyangiitis; MPA, microscopic polyangiitis; LAMP-2, lysosome associated membrane protein-2; MPO, myeloperoxidase; PAN, polyarteritis nodosa; PR3, proteinase 3; pVAS, pediatric vasculitis activity score; UCV, unclassified vasculitis; TOD, time of diagnosis.
