# Supplementary material for: Autoantibodies Against Lysosome Associated Membrane Protein-2 (LAMP-2) in Pediatric Chronic Primary Systemic Vasculitis
Source: Front Immunol. 2021 Feb 3;11:624758. doi: 10.3389/fimmu.2020.624758 (PMC7893977; doi:10.3389/fimmu.2020.624758)
Supplement: Supplementary file 1 [file DataSheet_1.pdf]

Supplementary Material

1 Supplementary Figures

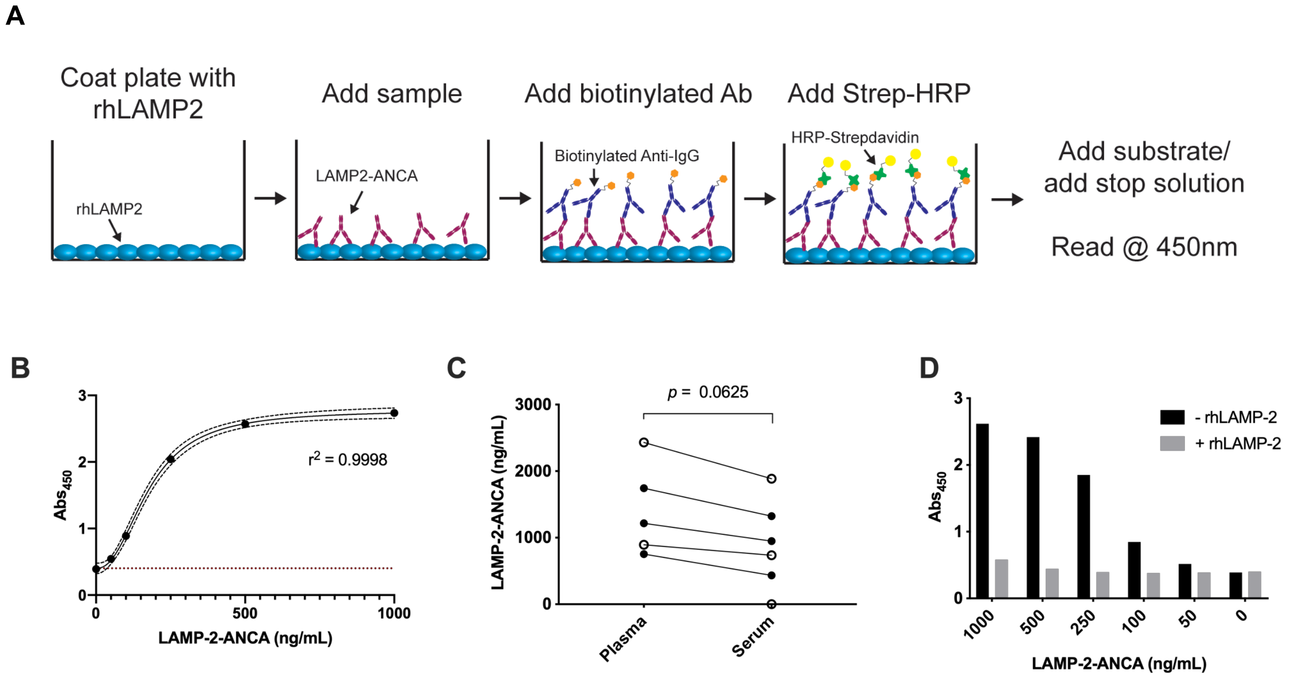

Supplementary Figure S1. **Custom indirect ELISA for detection of LAMP-2-ANCA in biosamples (serum or plasma). (A)** Schematic and **(B)** representative standard curve with serial dilutions of mouse anti-human LAMP-2 mAb (x-axis: 0, 50, 100, 250, 500, 1000 ng/mL) plotted against Absorbance at 450 nm (Abs_{450}; y-axis). Data points were fit to a sigmoidal, 4-parametric curve ($r^2 = 0.9998$). Dotted red line denotes lower-limit of detection ($Abs_{450} = 0.402$). **(C)** Concentration of LAMP-2-ANCA (y-axis; ng/mL) in paired plasma and serum (x-axis) from pediatric patients with vasculitis (n = 3; closed circles) and inflammation due to an autoinflammatory disease (n = 2; open circles). Paired plasma and sera from pediatric patients show slightly elevated LAMP-2-ANCA concentration in plasma (n = 5 $p = 0.0625$) **(D)** To validate the assay, standards (50 - 1000 ng/mL anti-human LAMP-2 mAb; x-axis) were added to the plate with (grey bars) and without (black bars) 25 µg rhLAMP-2. Absorbance (Abs_{450}; y-axis) of paired standards was measured. The rhLAMP-2 protein effectively blocks >97% of binding of 500 ng/mL mAb or less.
